# Factors Influencing the Surgical Decision in Dystonia Patients Referred for Deep Brain Stimulation

**DOI:** 10.3390/toxins13080511

**Published:** 2021-07-22

**Authors:** Carolina Gorodetsky, Paula Azevedo, Carolina Candeias da Silva, Alfonso Fasano

**Affiliations:** 1 Division of Neurology, The Hospital for Sick Children, Toronto, ON M5G 1X8, Canada; Carolina.gorodetsky@sickkids.ca; 2 Edmond J. Safra Program in Parkinson’s Disease, Morton and Gloria Shulman Movement Disorders Clinic, Toronto Western Hospital, UHN, Toronto, ON M5T 2S8, Canada; paulacazevedomov@gmail.com (P.A.); Carolina.CandeiasdaSilva@uhnresearch.ca (C.C.d.S.); 3 Division of Neurology, University of Toronto, Toronto, ON M5S 3H2, Canada; 4 Krembil Brain Institute, Toronto, ON M5T 1M8, Canada; 5 Center for Advancing Neurotechnological Innovation to Application (CRANIA), Toronto, ON M5T 1M8, Canada

**Keywords:** dystonia, deep brain stimulation, botulinum toxins, OnabotulinumtoxinA, IncobotulinumtoxinA, AbobotulinumtoxinA

## Abstract

There is no available data on the journey of dystonia patients once referred to a tertiary center to undergo deep brain stimulation (DBS). We hypothesized that some patients might be incorrectly diagnosed while others might decline the procedure or experience significant benefit with switching to a different botulinum neurotoxin (BoNT). This is a single-center, retrospective study of dystonia patients who were referred to the DBS program between January 2014 and December 2018. We collected data on the surgical decision as well as factors influencing this decision. Sixty-seven patients were included (30 males, mean age: 48.3 ± 20.1 years, disease duration: 16.9 ± 15.3 years). Thirty-three (49%) patients underwent DBS. Four (6%) patients were awaiting the procedure while the remaining 30 patients (45%) did not undergo DBS. Reasons for DBS decline were patient refusal (17, 53%), functional dystonia (6, 20%), and successful use of AbobotulinumtoxinA (3, 10%) in patients who had failed other BoNTs. Our study highlights the importance of structured patient education to increase acceptance of DBS, as well as careful patient evaluation, particularly with respect to functional dystonia. Finally, changing BoNT formulation might be beneficial in some patients.

## 1. Introduction

Dystonia is characterized by abnormal, often repetitive movements and/or postures that are caused by sustained or intermittent muscle contractions [1]. Patients with refractory dystonia are extremely challenging to treat and pose a significant burden to the medical system. There is no available data on the journey of these patients once referred to a tertiary center to undergo deep brain stimulation (DBS). For example, it is unknown what the proportion of patients eventually receiving surgery is and the reasons for exclusion, although similar studies have been carried out in other DBS indications such as Parkinson’s disease [2]. In addition, very little is known in terms of rescue treatments before confirming that dystonia is refractory to more conservative treatment.

Secondary non-response (SNR) is defined as the loss of a previous good clinical response to botulinum neurotoxin (BoNT). In the past, SNR was considered the result of neutralizing antibody against BoNT, but other factors—such as the underlying disease severity, were recently found to play a role in SNR [3]. Poor response to BoNT more frequently happens due to insufficient dose, inappropriate muscle selection and improper injection technique [4]. BoNT is the main modality of treatment in many of the focal and segmental dystonic conditions [5,6]; hence, the development of SNR can increase the need to refer these patients to more invasive treatments as DBS.

Of the two serotypes of BoNT used for dystonia treatment [5,6], in Canada, only serotype A is available. Three BoNT type A formulations are used in clinical practice: OnabotulinumtoxinA (Ona-A), IncobotulinumtoxinA (Inco-A) and the more recently approved AbobotulinumtoxinA (Abo-A). BoNT type B formulation (Rimabotulinumtoxinb, Rim-B) is not available in Canada. Despite the widespread use of different BoNTs in the dystonia population, there is a lack of comparative data on the efficacy of different formulations in patients with refractory dystonia, particularly the ones referred for DBS. Likewise, the role of changing BoNT type in patients with SNR is not established. Previous studies found immunogenicity ranges of 0.2% to 3.6% with Ona-A and 0.9% to 3.6% with Abo-A. Inco-A is less immunogenic formulation with immunogenicity ranging from 0 to 0.5%. The rates of neutralizing antibodies to Rim-B are higher than type A serotypes and ranges between 18 and 42.4%. The clinical relevance of these antibodies is uncertain [3,4] and the development and titer of these antibodies does not necessarily indicate the presence of SNR. Rim-B seems more likely to elicit SNR than BoNT-A [7].

We hypothesized that some patients deemed as ‘refractory’ by the referring physician are either not adequately treated (e.g., they might still experience significant improvements switching to a different BoNT) or not correctly diagnosed. Hence, the objective of this study was to evaluate the factors affecting the surgical decision in this population.

## 2. Results

### 2.1. Patient’s Demographics and Dystonia Classification

Sixty-seven (30 males, 45%) patients were included. The mean age at referral was 48.3 ± 20.1 years and mean disease duration was 16.9 ± 15.3 years. The majority (50, 75%) of the cohort had isolated dystonia. Generalized dystonia was the most common pattern of distribution (33%), followed by segmental (30%) and focal (22%) dystonia. A minority of patients presented with multi-focal (10%) and hemi-dystonia (4.5%). Seven (10%) patients had confirmed genetic diagnosis (including *THAP1, ADCY5, VPS41, ATXN3, AFG3L2, KMT2B*), 12 (18%) had acquired causes and the remainder had idiopathic dystonia. Five (7.5%) patients had a prior history of status dystonicus. (Table 1)

### 2.2. Oral Medications and BoNT Treatment Prior to the DBS Referral

At the time of referral, the majority of patients (49, 73%) were treated with at least one oral anti-dystonia medication, including baclofen, L-dopa, benzodiazepines, and anticholinergic medications (69% in the DBS group and 70% in the non-DBS group). Most of the patients (46, 69%) received BoNT treatment (Ona-A in 63%; Ona-A and Inco-A in 6%) for a duration of 6.1 ± 5.8 years and a mean number of BoNT sessions of 3.3 ± 1.3 in the year prior to referral. Fourteen patients (30%) had segmental dystonia, 13 (28%) focal dystonia, 13 (28%) generalized dystonia. The remainder of the patients had multifocal (4, 9%) and hemidystonia (2, 4%). A quarter of the injections were carried out with electromyography (EMG) assistance. None of the patients received Abo-A treatment prior to the referral. Twenty-four (52%) patients reported some benefit with BoNT and no side effects, but the benefit was only short lasting or unsatisfactory. Fifteen (33%) patients experienced no benefit and no side effects while the rest experienced bothersome side effects (7, 15%) including swallowing problems and muscle weakness.

### 2.3. Dystonia Severity during the Initial Assessment

TWSTRS data was available in 22 out of 31 patients with cervical dystonia (n: 15) and segmental dystonia involving the neck area (n: 16). The mean severity score was 14.95 ± 5.9; disability was 10.15 ± 6.2 and pain was 9.25 ± 4.6. The BFMDRS score was available for 16 out of 36 patients. The mean motor score was 54.0 ± 30.1 and disability was 18.25 ± 9.8.

### 2.4. DBS Selection Process 

**Figure 1:**
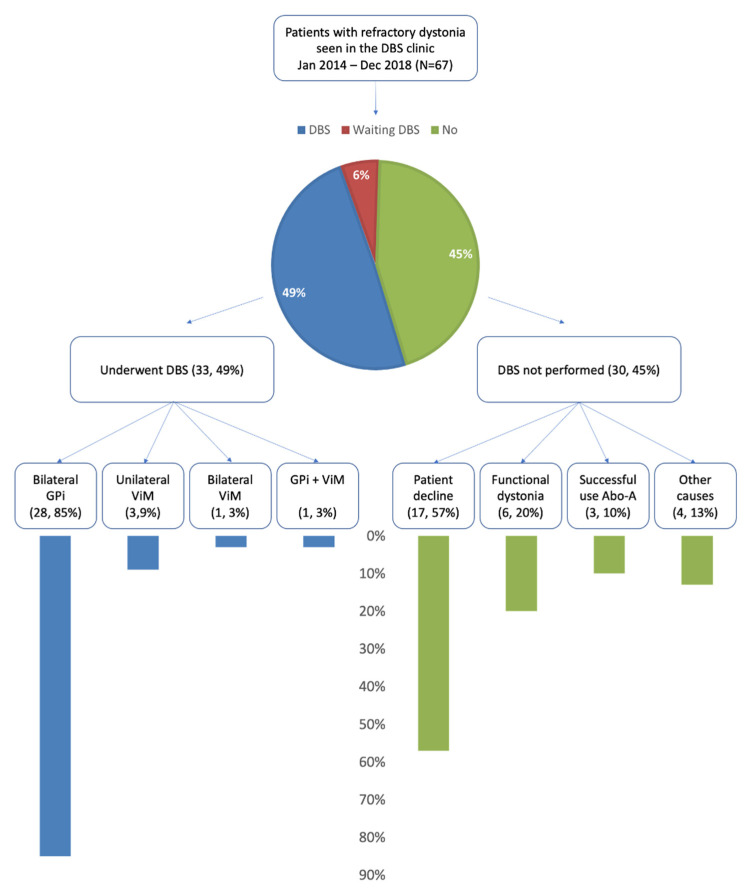
Flow diagram of dystonia patients who were seen in the DBS clinic. See text for details. Abbreviations: Abo-A—AbobotulinumtoxinA, DBS—deep brain stimulation, GPi—globus pallidus pars interna; ViM—ventral nucleus of the thalamus.

Thirty-three (49%) patients underwent DBS. Four (6%) patients were awaiting the procedure, while the remaining 30 (45%) patients did not undergo DBS (Figure 1).

#### 2.4.1. DBS Group

In the DBS group, twenty-eight (85%) underwent a procedure targeting the globus pallidus pars interna (GPi), three (9%) targeting the ventral nucleus of the thalamus (ViM), one (3%) underwent a staged bilateral ViM procedure and another case (3%) initially failed unilateral ViM and required a GPi procedure afterwards. Two patients underwent GPi twice due to device removal after an infection (Figure 1). We followed the DBS group for a mean duration of 3.9 ± 1.4 years.

#### 2.4.2. Non-DBS Group

The reasons for not performing DBS were: patient’s refusal (17, 57%), functional dystonia (6, 20%), and successful use of Abo-A (3, 10%) in patients who had failed other BoNTs. The remaining four patients (13%) were found not to be good DBS candidates because of a too mild condition (n: 1), presence of co-morbidities making DBS too risky (n: 1) or choice of unilateral MRI-guided focused ultrasound of the Vim (n: 2). The non-DBS group was followed for a mean duration of 1 ± 1.22 years.

##### Patients Who Declined DBS

Seventeen patients declined DBS surgery. Table 1 shows the dystonia distribution in the non-DBS and DBS groups. Most of the patients who declined surgery had segmental and focal dystonia (82% vs. 42% in the DBS group). We contacted these patients by phone to inquire about the reasons for their decision. The most common reason for declining DBS was patients concerns about the possible side effects and complications of the procedure (7, 44%). Two patients (12.5%) had doubts regarding the surgical outcomes, two more (12.5%) needed more time to decide and one patient (6%) improved with cannabidiol oil. Four patients were not available.

##### Functional Dystonia Patients

We identified a total of six patients who were referred for DBS consideration and were diagnosed with functional dystonia. The most common phenomenology was isolated segmental dystonia involving the neck as well as lower face and upper extremities. Table 2 details the demographic data of the functional cohort. Functional patients had a relatively short disease duration (7.8 ± 12.2 years) and only two (33.3%) were males. The diagnosis of functional dystonia was made according to a proposed standard diagnostic classification [8]: three patients were diagnosed as having a ‘clinically-definite’ functional movement disorder, two had a ‘clinically-established plus other features’ diagnosis and one patient had a ‘laboratory-supported definite’ diagnosis.

## 3. Discussion

We reported a longitudinal retrospective cohort of refractory dystonia patients who were referred for DBS consideration over a period of 5 years. Half of the cohort underwent DBS, with GPi being the most common target. In the non-DBS group, the main reasons for not performing DBS were patient refusal, diagnosis of functional dystonia and successful use of Abo-A toxin, not previously tried due to the recent approval in Canada. Our findings highlight the importance of providing a structured multidisciplinary patient education to improve the acceptance of DBS in this population, accurate diagnosis of dystonia (particularly excluding functional dystonia) as well as the possible benefit of switching BoNT formulation in patients refractory to other types.

### 3.1. Patient Education

The main reason for declining DBS in our population was patient refusal (57%), an unexpected finding never reported so far due to the lack of studies addressing the journey of dystonia patients refractory to conservative treatments. The most common reasons for the refusal were concerns of possible side effects and surgical complications. During the last decade, some studies have looked into the reasons for declining DBS in eligible patients with Parkinson’s disease [10]. The main reasons for DBS reluctance were a fear of complications and economic burden [11]. In one study, the use of a structured approach for patient’s pre-operative education informed the decision making and increased the DBS acceptance rate in Parkinson’s disease patients [10]. Comparable studies have not been conducted in dystonia patients, but not surprisingly, we found that similar factors play a role in the decision-making process. Of note, the economic burden would not apply to Canada because DBS is publicly funded without restrictions. It should, however, be noted that in our center we provide eligible DBS candidates with patient-centered written material (Appendix A) as well as in-person counseling. Developing a more structured and individualized multi-disciplinary approach might improve the attitude towards DBS in this population of refractory dystonia patients. Large prospective studies are needed to confirm this hypothesis.

### 3.2. Functional Dystonia

The diagnosis of functional dystonia can be very challenging. Core features include rapid onset with fixed posture at rest, distractibility, and absence of dystonia when the individual is unobserved [8,12]. In the absence of reliable lab-supported criteria (as seen in tremor patients), accurate diagnosis heavily relies on the expertise of movement disorder neurologist evaluating these patients. Our functional dystonia cohort was diagnosed based on the standard diagnostic classification and the majority of the patient (67%) were thought to have a definite functional disorder [8]. High vigilance for a functional diagnosis is recommended in this population. The treatment of functional dystonia involves a multidisciplinary approach with emphasis on psychotherapeutic interventions [13]. In fact, in addition to be exposed to an unnecessary surgical risk, patients with functional dystonia do not respond to DBS [14]. In our cohort, we identified six individuals (9% of all the dystonia patients who were referred for DBS consideration) with functional dystonia, in keeping with the notion that these patients enrich the cohorts of patients under consideration for DBS in light of their condition refractory to anti-dystonia drugs or BoNT. It should also be noted that Morigaki et al. [15] reported a cohort of five patients with combined ‘organic’ and functional dystonia who had a good response to DBS, making the distinction and final surgical decision even more complex.

### 3.3. Different BoNT Formulations in Refractory Dystonia

BoNT treatment for dystonia was initially introduced 30 years ago. There are three formulations of BoNT-A available in Canada (Ona-A, Inco-A and Abo-A). These formulations differ by methods of preparation, manufacturing as well as dosing [16]. Recent review provided level A evidence for BoNT-A efficacy in cervical dystonia [5,6,7], but the efficacy of this treatment in other forms of dystonia has not been established due to the rarity of these entities and the lack of clinical trials. Moreover, no studies were conducted to compare different BoNT-A formulations in refractory dystonia patients. The US Food and Drug administration approved Abo-A for treatment in adults with cervical dystonia in 2009 and similar approval was received by Health Canada in 2017. A recent systematic literature review established the safety, tolerability and efficiency of Abo-A in adult individuals with cervical dystonia [17]. A few head-to-head studies showed comparable efficacy in the treatment of cervical dystonia with Abo-A versus other BoNT-A formulations, mainly Ona-A and the Chinese BoNT-A (Institute of Biological Products, Lanzhou, China) [18,19,20]. Furthermore, a recent meta-analysis showed similar efficiency between Ona-A, Abo-A and Inco-A in cervical dystonia [21]. On the other hand, Abo-A was found to be more efficacious with longer benefit duration in the study by Ranoux et al. [22], who conducted a double-blind, randomized, cross-over study comparing two different doses of Abo-A to Ona-A in cervical dystonia patients. Similar studies were not conducted in a generalized dystonia population.

The majority of the patients who were referred to our DBS program had received prior treatments with BoNT-A (Ona-A and Inco-A), but these treatments were not sufficiently efficacious and/or produced bothersome side effects. Within the non-DBS group, 10% of patients experienced significant improvement of their symptoms after switching to a different formulation of BoNT-A, namely Abo-A. The decision regarding a trial of Abo-A before DBS was made collaboratively by the treating physician and the patient based on the fact that this was a new available formulation never tried at the time of the referral. We did not evaluate the presence of neutralizing antibodies in our cohort, although this occurrence is quite unlikely in our patients who responded to a different formulation of BoNT-A. In addition, previous studies showed that neutralizing antibodies are not the main cause for SNR and other factors such as insufficient dose, inappropriate muscle selection, improper injection technique as well as natural progression of the underlying disease play a very important role [3,4]. More importantly, an expert-based consensus paper suggests that SNR patients should be managed considering other options such as switching BoNT formulation or proposing DBS [7]. BoNT formulations are indeed different drugs and there might be unknown factors in determining patient’s responsiveness. We believe that a variable combination of these factors played a significant role in our patients who improved when using Abo-A.

### 3.4. Study Limitations

Our study has a few limitations. First of all, it carries all the limitations of a retrospective open-label study. Second, the refractory dystonia patients were seen in a single tertiary movement disorder center, thus meaning that our findings might not be generalized to a milder dystonia cases. Third, the decision regarding a trial of Abo-A before DBS was made collaboratively by the treating physician and the patient. Finally, the majority of referred patients were injected by other neurologists in different facilities, with different regimen and methods (e.g., EMG-guided treatment). However, these neurologists were movement disorders expert, who typically inject BoNT in Canada. In addition, in some cases, the same injector treated the same patient with different BoNTs obtaining different outcomes.

## 4. Conclusions

Our study—the first of this kind to our knowledge—highlights the importance of careful selection of dystonia patients for DBS, specifically excluding functional dystonia as well as providing comprehensive patient education and counseling prior to proceed with surgery. Additionally, some patients might experience a significant clinical benefit by switching BoNT formulation, thus avoiding the unnecessary risks of a neurosurgical intervention. Randomized double-blind controlled studies are needed to establish the best treatment in this interesting and selected population of patients with refractory dystonia.

## 5. Materials and Methods

This is a single-center, retrospective cohort study of children and adult dystonia patients who were referred to our DBS program between January 2014 and December 2018. This period was selected to have an adequate follow-up (at least 2 years) to determine the surgical decision and to allow the selected patients to undergo the surgical procedure.

Patients with other movement disorders were excluded. All the patients were referred to our clinic for DBS consideration by their primary provider (neurologist with expertise in movement disorders). They were informed about the main reason of the referral, but no details (type of surgery, outcomes, risks, etc.) were provided. All the patients underwent a standard clinical evaluation and received patient-oriented education material (Appendix A) from the DBS neurologist during the initial assessment. Some of the patients decided not to proceed with surgery after obtaining detailed information regarding the procedure. All the patients who were found to be good surgical candidates and were interested in the procedure underwent a standard multidisciplinary assessment that included clinical evaluation by a neurologist and a neurosurgeon with expertise in DBS. If clinically indicated, patients were assessed by the neuropsychologist and neuropsychiatrist of the multidisciplinary DBS team.

Demographic data, dystonia classification [1], BoNT formulations as well as anti-dystonia medications at the initial referral were collected. Neutralizing antibodies as well as other factors contributing to SNR were not assessed. There were no primary non-responders in our cohort. We also gathered data on the dystonia severity at the initial referral. TWSTRS (Toronto Western Spasmodic Torticollis Rating scale) [23] scores were documented for individuals with cervical dystonia and segmental dystonia involving the neck area while BFMDRS (Burke–Fahn–Marsden dystonia rating scale) [24] scores were used for the remainder of the cohort.

In the DBS group, we collected information regarding the surgical targets. In the non-DBS group, we contacted patients who refused DBS by phone to clarify the factors influencing their decision. Functional dystonia patients were diagnosed according to a proposed classification of functional movement disorders [8].

The study was conducted according to the guidelines of the Declaration of Helsinki and approved by the Institutional Research Board of the Toronto Western Hospital.

## Figures and Tables

**Table 1:** Patients’ demographics and clinical features at the initial assessment.

N=	67
Age (years ± SD)	48.3 ± 20.1
Disease duration (years ± SD)	16.9 ± 15.3
M:F	30:37
Dystonia distribution, DBS vs. non-DBS group:	
Generalized	22 (33%), 15 vs. 5 *
Segmental	20 (30%), 9 vs. 10 *
Focal	15 (22%), 5 vs. 9 *
Multi-focal	7 (10%), 2 vs. 5
Hemi-dystonia	3 (4.5%), 2 vs. 1
Dystonia etiology:	
Idiopathic	48 (72%)
Secondary	12 (18%)
Genetic	7 (10%)
BoNT treatment:	
Ona-A	42 (63%)
Ona-A + Inco-A	4 (6%)

Abbreviations: BoNT: botulinum neurotoxin, F: female, Inco-A: IncobotulinumtoxinA, M: male, Ona-A: OnabotulinumtoxinA, SD: standard deviation. * Four patients were awaiting the DBS procedure and were not included in the DBS and non-DBS groups (generalized dystonia (n − 2); focal (n − 1); segmental (n − 1)).

**Table 2:** Functional dystonia patients’ demographics and diagnostic classification.

N=	6
Age (years ± SD)	44 ± 16.7
M:F	2:4
Disease duration (years ± SD)	7.8 ± 12.2
Dystonia distribution:	
Segmental	50%
Focal	33%
Generalized	17%
BoNT treatment	83%
Diagnostic classification *	50% clinically definite 33% clinically established plus other features. 17% laboratory supported definite **.

Abbreviations: BoNT: botulinum neurotoxin, F: female, M: male, SD: standard deviation. * Diagnostic classification based on Gupta and Lang [8]. ** patient underwent EMG that showed absence of supportive criteria for organic dystonia as well as variable, inconsistent, distractable tremulous movements [9].

## Data Availability

The data presented in this study are available on request to the corresponding author.

## Appendix A

Toronto Western written material provided to patients prior to DBS surgery for dystonia.
